# Hemodynamic Profiles and Their Prognostic Relevance in Cardiac Amyloidosis

**DOI:** 10.3390/jcm9041093

**Published:** 2020-04-11

**Authors:** Franz Duca, Amir Snidat, Christina Binder, René Rettl, Theresa-Marie Dachs, Benjamin Seirer, Luciana Camuz-Ligios, Fabian Dusik, Christophe Denis Josef Capelle, Qin Hong, Hermine Agis, Renate Kain, Julia Mascherbauer, Christian Hengstenberg, Roza Badr Eslam, Diana Bonderman

**Affiliations:** 1 Department of Internal Medicine II, Department of Cardiology, Medical University of Vienna, 1090 Vienna, Austria; franz.duca@meduniwien.ac.at (F.D.); amir.snidat@meduniwien.ac.at (A.S.); christina.binder@meduniwien.ac.at (C.B.); rene.rettl@meduniwien.ac.at (R.R.); theresa-marie.dachs@meduniwien.ac.at (T.-M.D.); benjamin.seirer@meduniwien.ac.at (B.S.); luciana.camuzligios@meduniwien.ac.at (L.C.-L.); fabian.dusik@meduniwien.ac.at (F.D.); christophe.capelle@meduniwien.ac.at (C.D.J.C.); hong.qin@akhwien.at (Q.H.); julia.mascherbauer@meduniwien.ac.at (J.M.); christian.hengstenberg@meduniwien.ac.at (C.H.); 2 Department of Internal Medicine I, Department of Oncology, Medical University of Vienna, 1090 Vienna, Austria; hermine.agis@meduniwien.ac.at; 3 Clinical Institute of Pathology, Medical University of Vienna, 1090 Vienna, Austria; renate.agis@meduniwien.ac.at

**Keywords:** cardiac amyloidosis, pulmonary arterial pressures, invasive hemodynamics, outcomes

## Abstract

This study sought to characterize cardiac amyloidosis (CA) patients with respect to hemodynamic parameters and asses their prognostic impact in different CA cohorts. Intracardiac and pulmonary arterial pressures (PAPs) are among the strongest predictors of outcomes in patients with heart failure (HF). Despite that, the hemodynamic profiles of patients with CA and their relation to prognosis have rarely been investigated. Invasive hemodynamic, clinical, and laboratory assessment, as well as cardiac magnetic resonance imaging were performed in our CA cohort. A total of 61 patients, 35 (57.4%) with wild-type transthyretin amyloidosis (ATTRwt) and 26 (42.6%) with light-chain amyloidosis (AL) were enrolled. ATTRwt patients had lower N-terminal prohormone of brain natriuretic peptide values and were less frequently in New York Heart Association class ≥ III. Intracardiac and PAPs were elevated, but hemodynamic parameters did not differ between CA groups. Whereas in ATTRwt, the median mean PAP (hazard ratio (HR): 1.130, *p* = 0.040) and pulmonary vascular resistance (HR: 1.010, *p* = 0.046) were independent predictors of outcome, no hemodynamic parameter was associated with outcome in the AL group. Cardiac ATTRwt and AL patients feature elevated intracardiac and PAPs and show similar hemodynamic profiles. However, hemodynamic parameters are of greater prognostic relevance in ATTRwt, potentially providing a new therapeutic target.

## 1. Introduction

Once deemed an ultra-rare disease, cardiac amyloidosis (CA) is now considered an important etiology in the diagnostic work-up of heart failure (HF) and an increasing number of cardiologists are confronted with the difficult task of treating these patients [1,2]. In HF, irrespective of left ventricular ejection fraction (LVEF), intracardiac filling pressures and pulmonary arterial pressures (PAPs) play a central pathophysiological role and are among the strongest predictors of adverse outcomes [3,4]. Supporting the importance of hemodynamics in HF, the recently published phase III VerICiguaT Global Study in Subjects with Heart Failure With Reduced Ejection Fraction (VICTORIA) trial, which tested vericiguat, a soluble guanylate cyclase stimulator that acts as a vasodilator in the pulmonary and systemic circulation, has met the primary endpoint i.e., the time to the first occurrence of cardiovascular death or HF hospitalization [5].

Contrary to HF, data on hemodynamics in CA are rare, stem from retrospective studies and provide conflicting results [6,7]. Therefore, further research is warranted to investigate hemodynamic profiles in different CA subgroups and to assess whether intracardiac filling pressures and PAPs are associated with patient outcomes, thus providing potential new therapeutic targets in CA.

Hence, we aimed to characterize CA patients with respect to invasively measured hemodynamic parameters and assess their prognostic relevance in a prospectively followed cohort consisting of wild-type transthyretin amyloidosis (ATTRwt) and light-chain amyloidosis (AL) patients.

## 2. Materials and Methods

### 2.1. Setting and Study Design

The present study was performed within the scope of a prospective HF registry at the Department of Internal Medicine II, Division of Cardiology at the Medical University of Vienna, which includes a dedicated amyloidosis outpatient clinic. The study was approved by the local ethics committee (EK# 796/2010) and conducted according to good clinical practice as outlined in the declaration of Helsinki. All patients gave written informed consent.

### 2.2. Clinical Definitions

#### 2.2.1. Diagnosis of Cardiac Transthyretin Amyloidosis

Until June 2016, cardiac transthyretin amyloidosis (ATTR) was diagnosed if endomyocardial biopsy (EMB) samples stained positive for Congo red, showed apple green birefringence under polarized light and reacted with anti-TTR antibodies during immunohistochemical staining. Following the publication of Gillmore and colleagues in June 2016, who provided an algorithm that allows a non-biopsy diagnosis of cardiac ATTR [8], obtaining EMBs became only necessary if non-invasive test results, including cardiac magnetic resonance imaging (CMRi), transthoracic echocardiography (TTE), bone scintigraphy, serum immunofixation, urine immunofixation, and serum free light-chain assay were ambiguous. If cardiac ATTR, irrespective of diagnostic algorithm, was diagnosed, patients were offered sequencing of the TTR gene.

#### 2.2.2. Diagnosis of Cardiac Light-Chain Amyloidosis

Cardiac AL was diagnosed if EBM or extra-myocardial biopsy samples stained positive for Congo red, showed apple green birefringence under polarized light and reacted with light-chain antibodies during immunohistochemical staining. In order to confirm cardiac involvement in the case of extra-myocardial biopsy, we complied with current recommendations [9].

#### 2.2.3. Obtaining and Histological Analysis of Biopsy Samples

EMB specimens were acquired from the left ventricle during left heart catheterization (Bipal^®^ biopsy forceps, Cordis^®^ Corporation, Bridgewater, NJ, USA). All biopsy specimens, irrespective of biopsy site, were immediately fixed in 7.5% buffered formalin for 24 h followed by paraffin embedding. Samples were cut in 2 µm, 3 µm and 6 µm sections using a Leica RM 2255 Microtome (Charleston, SC, USA). Slices of 3 µm were stained with modified trichrome [10], 6 µm sections were used for Congo red staining, and visualization under polarized light. Slices of 2 µm were used for immunohistochemical staining (AmY-kit amyloid antibodies, Martinsried, Germany).

### 2.3. Cardiac Magnetic Resonance Imaging

CMRi was performed on a 1.5 Tesla scanner (MAGNETOM Avanto, Siemens Healthcare GmbH, Erlangen, Germany) according to standard protocols [11], which included late gadolinium enhancement imaging (0.1 mmoL/kg gadobutrol, Gadovist, Bayer Vital GmbH, Leverkusen, Germany) and T1 mapping using the modified Look–Locker inversion (MOLLI) sequence [11,12,13]. 

#### 2.3.1. Transthorathic Echocardiography

TTE was performed by certified and experienced operators on high-end machines (GE Vivid E95 and Vivid 7; GE Healthcare, Wauwatosa, WI, USA) according to current recommendations [14,15]. Left-sided heart valve stenosis or regurgitation ≥ moderate was deemed significant.

#### 2.3.2. Invasive Hemodynamic Assessment

Hemodynamic parameters were assessed during right heart catheterization with a 7F Swan-Ganz catheter (Edwards Lifesciences, Irvine, CA, USA). Average pressures over eight heart cycles were used for analysis (CathCorLX (Siemens AG, Berlin and Munich, Germany)). Directly measured parameters were mean PAP (mPAP), pulmonary artery wedge pressure (PAWP), right atrial pressure (RAP), cardiac index (CI), and stroke volume index (SVi). Further parameters of interest, which included diastolic pressure gradient and pulmonary vascular resistance (PVR), were calculated according to standard formulae [16]. 

### 2.4. Outcome Measures

The primary outcome measure was a combination of cardiovascular death and hospitalization for HF.

### 2.5. Statistical Analysis

Continuous variables are expressed as the median and interquartile range (IQR). Categorical variables are presented as numbers and percentages. Continuous and categorical variables were compared using the Mann–Whitney U test and chi-square test as appropriate. For all tests, the significance level was set to *p* < 0.05.

To assess the effect of parameters of interest on event-free survival, separate uni- and multivariable Cox regression models were calculated for clinical, invasive hemodynamic, and CMRi parameters. In order to account for the limited number of events, we did not perform stepwise Cox regression analyses, but adjusted for the N-terminal prohormone of brain natriuretic peptide (NT-proBNP) and troponin t, which are well-established predictors of adverse outcomes in CA [17,18,19].

Kaplan–Meier plots (log rank test) were used to verify the time-dependent discriminative power of parameters of interest. Spearman correlation coefficients were used for correlation analyses. International Business Machines Corporation (IBM) SPSS version 26.0 (IBM Corp. Chicago, United States of America) was used for all statistical analyses.

## 3. Results

### 3.1. Baseline Characteristics and Clinical Presentation of the Overall Cohort

**Figure 1:**
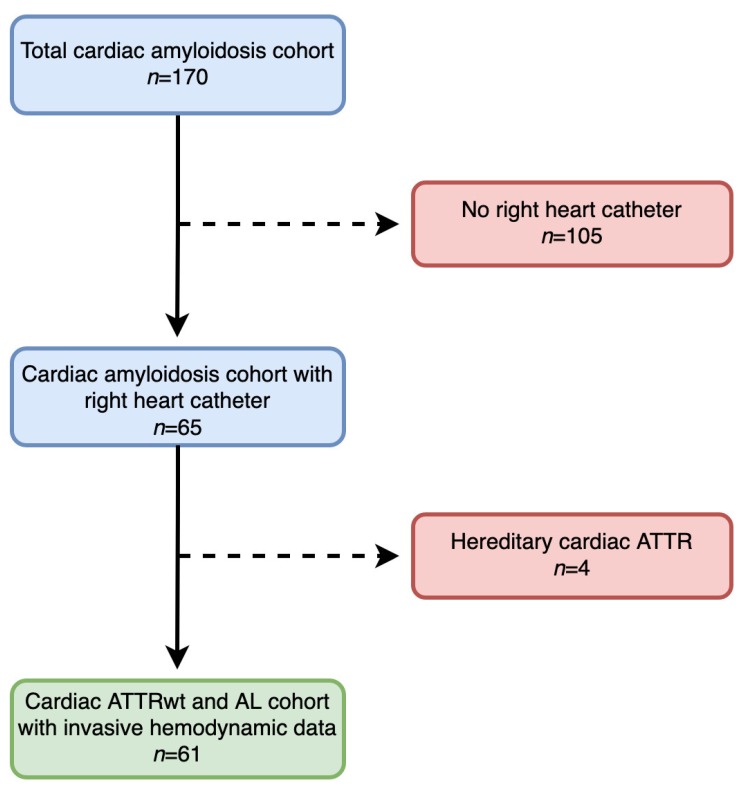
Patient flow-chart. A total of 170 patients were screened for the study. Reasons for exclusion are displayed. Transthyretin amyloidosis (ATTR); wild-type transthyretin amyloidosis (ATTRwt); light-chain amyloidosis (AL).

Between March 2012 and January 2019, 170 patients with CA were included into our prospective registry. Of those, 109 had to be excluded from our analysis, the main reason being unwillingness to undergo invasive hemodynamic assessment. A detailed patient flowchart is provided in Figure 1.

Eventually, 61 CA patients who underwent invasive hemodynamic assessment were eligible for final data analysis, of whom 35 (57.4%) patients were diagnosed with ATTRwt and 26 (42.6%) with AL. Baseline characteristics are shown in Table 1.

Median NT-proBNP values of 3552 pg/mL (IQR: 1501–7357) and the fact that almost half (*n* = 30, 49.2%) of all patients were in New York Heart Association (NYHA) class ≥ III suggest that study participants were in rather advanced stages of HF. No difference with respect to HF severity could be detected between patients who underwent invasive hemodynamic assessment and patients who did not (NYHA class ≥ III: *n* = 30.0 (49.2) % vs. *n* = 38.0 (36.2%), *p* = 0.106; median NT-proBNP: 3552 pg/mL (IQR: 1501–7357) versus 2972 pg/mL (IQR: 1238–6801), *p* = 0.478).

Furthermore, patients showed marked elevations in pulmonary arterial and intracardiac filling pressures (mPAP: 30.0 mmHg, IQR: 25.5–36.5; RAP: 11.0 mmHg, IQR: 7.3–16.8; PAWP: 20.0 mmHg, IQR: 16.5–24.0), whereas CI (2.4 L/min/m^2^, IQR: 1.9–2.8) and SVi (30.7 mL/m^2^, IQR: 25.2–41.6) were mostly within normal ranges.

Regarding cardiac structural and functional parameters, patients had pronounced left ventricular (LV) hypertrophy (interventricular septum (IVS): 19 mm, IQR: 15.5–22.0) and elevated MOLLI-extracellular volume (MOLLI-ECV) values (47.2%, IQR: 41.0–55.9). LVEF, LV end-diastolic volume index (EDVi), right ventricular (RV) EF, and RVEDVi were 57.5% (IQR: 50.0–66.3), 66.1 mL/m^2^ (IQR: 99.8–170), 48.0% (IQR: 41.0–61.5), and 78.5 mL/m^2^ (IQR: 64.0–96.7), respectively.

Significant combined aortic valve disease was present in one patient (1.6%). Relevant mitral regurgitation (MR) was present in 20 (32.8%) patients.

### 3.2. Differences Between Cardiac Wild-Type Transthyretin and Light-Chain Amyloidosis Patients

We detected clear clinical differences between cardiac ATTRwt and AL patients (Table 1). ATTRwt patients were older (75.0 years, IQR: 73.0–82.0 versus 68.0 years, IQR: 54.8–74.3; *p* < 0.001), more often male (*n* = 28, 80% vs. *n* = 10, 38.5; *p* = 0.001), less symptomatic (NYHA class ≥ III: *n* = 14, 40% versus *n* = 16, 61.5%; *p* = 0.027), and had lower levels of NT-proBNP (2368 pg/mL, IQR: 1331–5834 vs. 4900 pg/mL, IQR: 2045–10,571; *p* = 0.020).

Invasive hemodynamic parameters, however, did not differ between CA subtypes. Differences with regards to imaging parameters are provided in Table 1.

### 3.3. Patient Outcomes

During a median follow-up of 19.0 months (IQR: 3.5–32.0), 30 patients (49.2%) reached the composite endpoint of either HF hospitalization or cardiovascular death (Table 1). Median overall survival in the total CA cohort was 49.0 months.

Compared to AL patients, ATTRwt patients experienced the endpoint less often (*n* = 10, 28.6% versus *n* = 20, 76.9%; *p* < 0.001). Median overall survival was 14.0 months in the AL and 53.0 months in the ATTRwt cohorts.

Uni- and multivariable Cox regression analyses and Kaplan–Meier curves for the total CA cohort are shown in Table S1 and Figure S1.

Clinical parameters that were independently associated with outcome were age (hazard ratio (HR): 0.958, 95% confidence interval (CI): 0.924–0.993; *p* = 0.020), male gender (HR: 0.277, 95% CI: 0.122–0.628; *p* = 0.002), NYHA class ≥ III (HR: 3.214, 95% CI: 1.211–8.529; *p* = 0.019), NT-proBNP (HR: 1.940, 95% CI: 1.327–2.838; *p* = 0.001), and troponin t (HR: 1.558, 95% CI: 1.073–2.264; *p* = 0.020). Among hemodynamic parameters mPAP (HR: 1.034, 95% CI: 1.001–1.069; *p* = 0.045) and PAWP (HR: 1.073, 95% CI: 1.011–1.139; *p* = 0.021) remained predictors of outcome in our multivariable model. LVEDVi (HR: 0.972, 95% CI: 0.952–0.992; *p* = 0.007) was the only CMRi parameter predictive of adverse events. No association between patient outcomes and concomitant valvular heart disease was found [aortic stenosis (AS): HR: 1.072, 95% CI: 0.142–8.109; *p* = 0.946; aortic regurgitation (AR): HR: 1.072, 95% CI: 0.142–8.109; *p* = 0.946, MR: HR: 1.116, 95% CI: 0.529–2.353; *p* = 0.773).

### 3.4. Predictors of Outcome in Cardiac Wild-Type Transthyretin Amyloidosis Patients

Uni- and multivariable Cox regression analyses for ATTRwt patients are shown in Table 2. Among clinical parameters, age (HR: 1.255, 95% CI: 1.055–1.493; *p* = 0.010], diastolic blood pressure (HR: 0.897, 95% CI: 0.828–0.971; *p* = 0.008), troponin t (HR: 2.466, 95% CI: 1.187–5.124; *p* = 0.016), and the number of diuretic agents (HR: 5.932, 95% CI: 1.217–28.90; *p* = 0.028) were independent predictors of adverse patient outcomes.

Among invasive hemodynamic parameters, mPAP (HR: 1.130, 95% CI: 1.006–1.269; *p* = 0.040) and PVR (HR: 1.010, 95% CI: 1.000–1.020; *p* = 0.046) were associated with outcomes in the multivariable analyses.

MOLLI-ECV was predictive of outcomes in the univariable, but not in the multivariable analyses.

**Figure 2:**
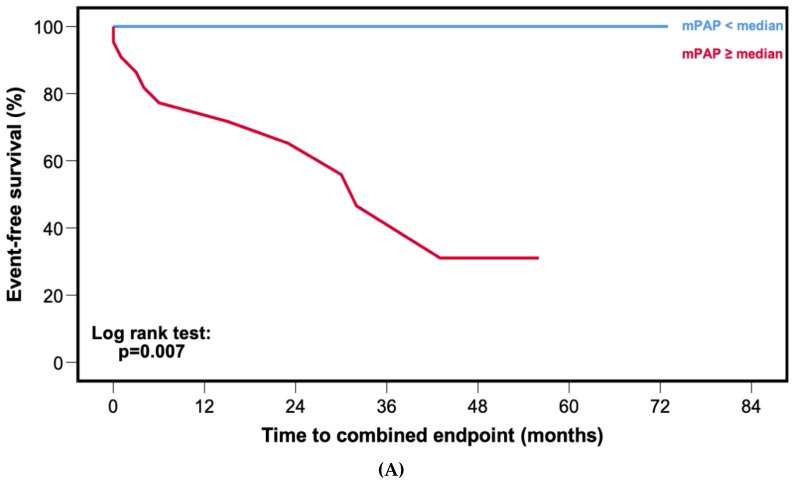

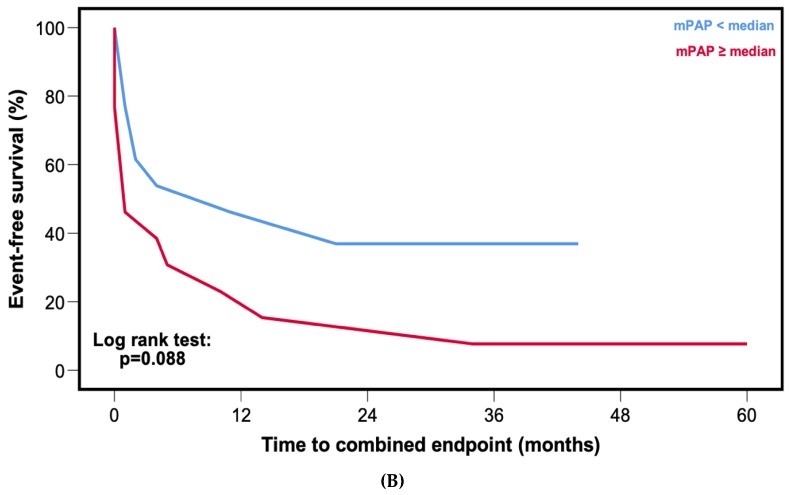
Kaplan–Meier curves for event-free survival stratified by median mean pulmonary arterial pressure (mPAP). (**A**) Patients with cardiac wild-type transthyretin amyloidosis. (**B**) Patients with cardiac light-chain amyloidosis.

Kaplan–Meier curves (Figure 2A) and corresponding log rank test (*p* = 0.007) showed worse event-free survival for patients with mPAP ≥ median (30.0 mmHg).

### 3.5. Predictors of Outcome in Cardiac Light-Chain Amyloidosis Patients

Uni- and multivariable Cox regression analyses for cardiac AL patients are depicted in Table 3.

Despite the fact that a series of parameters, such as NYHA class ≥ III, systolic blood pressure, troponin t, SVi, MOLLI-ECV, LVEDVi, and IVS were associated with outcomes in the univariable Cox regression model, NT-proBNP (HR: 2.856, 95% CI: 1.277–6.388; *p* = 0.011) was the only parameter that remained predictive of outcomes in the multivariable Cox regression analyses.

In contrast to ATTRwt, there was no statistical difference in event-free survival among patients with mPAP ≥ median (32.0 mmHg) compared to patients with mPAP < median (Figure 2B, *p* = 0.088).

### 3.6. Comparison of Outcomes in Cardiac Wild-Type Transthyretin and Light-Chain Amyloidosis

In a further analysis, we compared ATTRwt patients with mPAP ≥ and < median against AL patients with mPAP ≥ and < the median and could detect significant differences in event-free survival (graphical abstract, *p* < 0.001). AL patients with mPAP ≥ median had the worst event-free survival, whereas ATTRwt patients with mPAP < median had the best event-free survival. Interestingly, there was no difference in event-free survival between patients with ATTRwt and median ≥ mPAP and AL patients < median mPAP.

### 3.7. Association of Pulmonary Arterial Pressures with Valvular Heart Disease and Diuretics

In the total CA cohort, there was no association between the presence of valvular heart disease and mPAP or PAWP (r = 0.008, *p* = 0.954 for AS and mPAP; r = 0.064, *p* = 0.627 for AS and PAWP; r = 0.008, *p* = 0.954 for AR and mPAP; r = 0.064, *p* = 0.627 for AR and PAWP; r = 0.110, *p* = 0.401 for MR and mPAP; r = 0.216, *p* = 0.098 for MR and PAWP). However, a clear association between the number of diuretic agents (r = 0.342, *p* = 0.007) and mPAP levels could be detected.

## 4. Discussion

The present study assessed the prognostic relevance of intracardiac and PAPs in a well-characterized, prospectively followed cohort of CA patients. The major findings of our study were that (1) ATTRwt and AL patients do not differ with respect to hemodynamics, but (2) hemodynamic parameters are of greater prognostic importance for ATTRwt patients compared to AL.

### 4.1. Hemodynamic Profiles in Cardiac Amyloidosis

Despite their prognostic significance in other types of HF, such as HF with preserved and reduced LVEF, data on invasively measured hemodynamics in CA patients are scarce [6,20,21].

In line with our results, a retrospective study by Russo et al. could detect similar hemodynamics between cardiac ATTR and AL patients [6]. Furthermore, mean intracardiac filling pressures were comparable in their and our CA patient cohorts. In contrast to our study, they found that RAP was the strongest predictor of adverse patient outcomes. One possible explanation for this difference might simply be the fact that mPAP, the strongest outcome predictor among hemodynamic parameters in our series, was not measured in the aforementioned study [6]. Also, they did not perform separate survival analyses for CA subgroups.

In a further study, hemodynamic profiles differed between AL and ATTR patients, with lower RAP and PAWP values in the ATTR group [7]. However, compared with our cohort, ATTR patients were in less advanced stages of the disease, as suggested by less pronounced LV hypertrophy.

### 4.2. Pulmonary Arterial Pressures as a Potential Therapeutic Target in Cardiac Amyloidosis

One of the major findings in the present study was the difference in the prognostic relevance of mPAP between ATTRwt and AL. In our ATTRwt patients, mPAP was the strongest predictor of outcomes among invasively obtained hemodynamic parameters (Table 2, HR: 1.130, 95% CI: 1.006–1.269; *p* = 0.040). Interestingly, when patients were stratified according to median mPAP (30.0 mmHg), Kaplan–Meier curves showed that not a single patient with mPAP < median experienced an outcome event (Figure 2A, log rank: *p* = 0.007). In contrast, among AL patients mPAP was neither associated with adverse outcome in the Cox regression (Table 3, HR: 1.027, 95% CI: 0.991–1.063; *p* = 0.146) nor in the Kaplan–Meier analysis (Figure 2B, log rank: *p* = 0.088). Furthermore, ATTRwt patients with mPAP ≥ median had comparable outcomes to patients with AL and mPAP < median (Graphical abstract).

Given its prognostic significance, PAPs could provide an intriguing therapeutic target in this difficult-to-treat patient population. The pathophysiological mechanism behind CA is the deposition of amyloid in the cardiac extracellular matrix. This leads to the thickening, stiffening, and subsequent diastolic dysfunction of the LV. The deterioration of LV diastolic function then causes a rise in left-sided intracardiac filling pressures and PAPs, which increases the afterload posed on the RV. A reduction of PAPs decreases the load on the RV, thus improving RV function, which in turn is strongly associated with prognosis in cardiac ATTR, but not AL patients [22]. However, this hypothesis needs to be tested in interventional trials. A potential class of drugs that might be used in this setting could be soluble guanylate cyclase stimulators, which are successfully used in the treatment of precapillary forms of pulmonary hypertension, but can be administered safely in HF, and as suggested by a small case series from our study group, also in cardiac ATTR patients [23,24,25]. However, one major limitation is that this class of drugs does not stop amyloid from being produced or expedite its removal, thus not targeting the underlying pathophysiological mechanism in CA.

In a recently published phase III trial, administration of Tafamidis, a stabilizer of the TTR tetramer and one of the most promising drugs in cardiac ATTR, significantly reduced all-cause mortality as well as cardiovascular-related hospitalizations [26]. Of note was the fact that survival curves only started to diverge at 18 months of treatment. Therefore, optimizing PAPs could have an additional effect as more patients reach the timepoint where the survival benefits of amyloid-specific therapies become evident.

### 4.3. Association of Diuretics with Pulmonary Arterial Pressures and Outcome

We observed that the number of diuretics was correlated with mPAP (r = 0.342, *p* = 0.007) and was predictive of outcome in ATTRwt (HR: 5.932, 95% CI: 1.217–28.906; *p* = 0.028), but not in the AL cohort (HR: 1.456, 95% CI: 0.777–2.720, *p* = 0.241). As the use of diuretics represents a surrogate for overhydration and more advanced HF stages, our findings are in line with earlier studies showing that, in contrast to AL, the most common mode of death in ATTRwt patients is cardiovascular [27].

### 4.4. Limitations

Several limitations are inherent to the present study, such as the single-center design. However, this study design allows for constant diagnostic work-up and clinical follow-up of patients. Moreover, the invasive design of our study might have led to the selection of patients in more advanced stages of HF. Nevertheless, there was no difference in NYHA class or NT-proBNP values between patients who underwent invasive hemodynamic assessment to those who have declined. Further limitations are the sample size of our cohort and the limited number of outcome events, both of which do not allow extensive Cox regression analyses. Thus, our results must be interpreted with caution, as low event per variable ratios may cause type I errors and bias away from the null hypothesis. Nevertheless, the validity of our Cox regression model is supported by numerically stable hazard ratio estimates and 95% CIs. In addition, our cohort was in a rather advanced stage of HF, potentially further limiting our outcome analyses. However, the outcome of our cardiac ATTRwt and AL cohorts resemble contemporary CA cohorts from other centers [28,29]. Due to the fact that our study cohort consists only of cardiac ATTRwt and AL patients, the generalizability of our data to other forms of CA, especially hereditary ATTR, is limited. However, we decided to exclude patients with mutations in the TTR gene from our analysis (*n* = 4, Figure 1) because this patient population is highly heterogenous and has a vastly different course of disease compared to ATTRwt.

## 5. Conclusions

In this prospective registry study, we investigated the hemodynamic profiles of patients with cardiac ATTRwt and AL. Intracardiac and PAPs were elevated to a similar extent in both forms of CA.

The main finding of our study, however, was that cardiac hemodynamics were strong predictors of outcomes in ATTRwt, but not in AL, thus potentially providing a new therapeutic target in these difficult-to-treat patients with whose treatment more and more cardiologists are confronted on a regular basis. Nonetheless, given the limitations, our results must be interpreted very cautiously and studies of stronger design are warranted to further evaluate the clinical importance of our findings.

## Figures and Tables

**Table 1:** Baseline characteristics.

Variable	All Patients (*n* = 61)	ATTRwt (*n* = 35)	AL (*n* = 26)	*p* Value
**Clinical parameters**				
**Age, years (IQR)**	74.0 (66.5–78.0)	75.0 (73.0–82.0)	68.0 (54.8–74.3)	**<0.001**
**Sex, male gender, *n* (%)**	38.0 (62.3)	28.0 (80.0)	10.0 (38.5)	**0.001**
**NYHA functional class ≥ III, *n* (%)**	30.0 (49.2)	14 (40.0)	16.0 (61.5)	**0.027**
Systolic blood pressure, mmHg (IQR)	118 (112–139)	122 (114–135)	117 (103–142)	0.431
Diastolic blood pressure, mmHg (IQR)	68.0 (61.0–78.0)	67.0 (61.0–74.5)	70.0 (63.8–78.3)	0.521
**NT-proBNP, pg/mL (IQR)**	3552 (1501–7357)	2368 (1331–5834)	4900 (2045–10571)	**0.020**
Troponin t, ng/mL (IQR)	0.07 (0.03–0.12)	0.07 (0.03–0.10)	0.07 (0.03–0.21)	0.611
eGFR, mL/min/1.73 m^2^ (IQR)	58.4 (43.8–74.8)	56.4 (43.5–79.6)	61.2 (46.2–71.3)	0.838
**Combined endpoint, *n* (%)**	30.0 (49.2)	10.0 (28.6)	20.0 (76.9)	**<0.001**
**Medication**				
Beta Blocker, *n* (%)	34.0 (55.7)	19.0 (54.3)	15.0 (57.7)	0.889
ACE inhibitor, *n* (%)	14.0 (23.0)	8.0 (22.9)	6.0 (23.1)	0.967
Angiotensin receptor blocker, *n* (%)	17.0 (27.9)	11.0 (31.4)	6.0 (23.1)	0.429
Epigallocatechin gallate, *n* (%)	20.0 (32.8)	20.0 (57.1)	0.0 (0.0)	n.a
Tafamidis, *n* (%)	2.0 (3.3)	2.0 (5.7)	0.0 (0.0)	n.a
Daratumumab, *n* (%)	5.0 (8.2)	0.0 (0.0)	5.0 (19.2)	n.a
Lenalidomide, *n* (%)	1.0 (1.6)	0.0 (0.0)	1.0 (3.8)	n.a
Thalidomide, *n* (%)	2.0 (3.3)	0.0 (0.0)	2.0 (7.7)	n.a
Bortezomib, *n* (%)	14.0 (23.0)	0.0 (0.0)	14.0 (53.8)	n.a
Cyclophosphamide, (%)	8.0 (13.1)	0.0 (0.0)	8.0 (30.8)	n.a
Dexamethasone, *n* (%)	14.0 (23.0)	0.0 (0.0)	14.0 (53.8)	n.a
Rituximab, *n* (%)	1.0 (1.6)	0.0 (0.0)	1.0 (3.8)	n.a
Bendamustine, *n* (%)	1.0 (1.6)	0.0 (0.0)	1.0 (3.8)	n.a
No diuretic agents, *n* (%)	12.0 (19.7)	8.0 (22.9)	4.0 (15.4)	0.434
One diuretic agent, *n* (%)	13.0 (21.3)	10.0 (28.6)	3.0 (11.5)	0.096
Two diuretic agents, *n* (%)	28.0 (45.9)	13.0 (37.1)	15.0 (57.7)	0.134
Three diuretic agents, *n* (%)	7.0 (11.5)	3 (8.6)	4.0 (15.4)	0.433
**Invasive hemodynamic parameters**				
Mean pulmonary arterial pressure, mmHg (IQR)	30.0 (25.5–36.5)	30.0 (26.0–34.0)	32.0 (25.0–43.0)	0.296
Right atrial pressure, mmHg (IQR)	11.0 (7.3–16.8)	11.0 (7.8–16.0)	11.5 (7.0–18.0)	0.654
Pulmonary artery wedge pressure, mmHg (IQR)	20.0 (16.5–24.0)	19.0 (16.0–22.0)	20.5 (16.8–29.3)	0.201
Cardiac index, L/min/m^2^ (IQR)	2.4 (1.9–2.8)	2.4 (2.0–2.7)	2.4 (1.8–3.1)	0.941
Stroke volume index, mL/m^2^ (IQR)	30.7 (25.2–41.6)	31.4 (24.4–42.3)	30.3 (25.9–40.2)	0.835
Pulmonary vascular resistance, dyn·s·cm^−5^ (IQR)	180 (129–266)	181 (128–300)	166 (126–264)	0.726
Diastolic pressure gradient, mmHg (IQR)	1.0 (−1.0–3.8)	2.0 (−1.0–4.3)	0.0 (−1.3–3.0)	0.217
**Cardiac magnetic resonance imaging parameters**				
MOLLI-ECV, % (IQR)	47.2 (41.0–55.9)	48.0 (41.1–55.6)	45.8 (39.6–65.4)	0.860
Left atrial area, cm^2^ (IQR)	31.5 (26.0–37.3)	32.5 (27.8–38.8)	30.0 (24.0–31.0)	0.185
**Right atrial area, cm^2^ (IQR)**	29.0 (24.0–38.0)	33.0 (27.8–39.5)	25.5 (24.0–31.0)	**0.010**
Left ventricular ejection fraction, % (IQR)	57.5 (50.0–66.3)	55.6 (49.0–60.5)	62.5 (52.3–67.0)	0.077
**Left ventricular end-diastolic volume index, ml/m^2^ (IQR)**	66.1 (56.5–85.0)	81.6 (64.9–91.5)	60.1 (45.9–72.3)	**0.001**
**Interventricular septum, mm (IQR)**	19.0 (15.5–22.0)	20.0 (16.0–23.0)	17.0 (13.0–20.0)	**0.040**
Right ventricular ejection fraction, % (IQR)	48.0 (41.0–61.5)	48.0 (38.0–60.5)	52.0 (42.0–62.0)	0.629
Right ventricular end-diastolic volume index, mL/m^2^ (IQR)	78.5 (64.0–96.7)	82.8 (66.7–100)	72.3 (62.1–94.0)	0.133
**Transthorathic echocardiography parameters**				
Significant aortic valve stenosis, *n* (%)	1.0 (1.6)	1.0 (2.9)	0.0 (0.0)	0.378
Significant aortic valve regurgitation, *n* (%)	1.0 (1.6)	1.0 (2.9)	0.0 (0.0)	0.378
Significant mitral valve stenosis, *n* (%)	0.0 (0.0)	0.0 (0.0)	0.0 (0.0)	n.a
Significant mitral valve regurgitation, *n* (%)	20.0 (32.8)	12.0 (34.3)	8.0 (30.8)	0.713

Wild-type transthyretin amyloidosis (ATTRwt); light-chain amyloidosis (AL); interquartile range (IQR); New York Heart Association (NYHA); N-terminal prohormone of brain natriuretic peptide (NT-proBNP); estimated glomerular filtration rate (eGFR); angiotensin converting enzyme (ACE); modified Look–Locker inversion recovery sequence derived extracellular volume (MOLLI-ECV). Valvular stenosis or regurgitation ≥ moderate was considered significant; n.a indicates not applicable. Bold indicates statistical significance.

**Table 2:** Cox regression analysis for the cardiac wild-type transthyretin amyloidosis cohort.

Variable	Crude Hazard Ratio	95% Confidence Interval	*p* Value	Adjusted Hazard Ratio	95% Confidence Interval	*p* Value
	Univariable Regression			Multivariable Regression		
**Clinical parameters**						
**Age, years**	1.143	1.021–1.279	**0.020**	1.255	1.055–1.493	**0.010**
Sex, male gender	2.252	0.284–17.843	0.442	0.387	0.025–5.987	0.497
NYHA functional class ≥ III	1.441	0.379–5.485	0.592	2.927	0.662–12.931	0.157
Systolic blood pressure, mmHg	0.990	0.955–1.027	0.602	0.985	0.954–1.017	0.353
**Diastolic blood pressure, mmHg**	0.966	0.906–1.029	0.283	0.897	0.828–0.971	**0.008**
NT-proBNP, pg/mL*	1.383	0.769–2.487	0.279	1.181	0.641–2.176	0.593
**Troponin t, ng/mL †**	2.499	1.231–5.072	**0.011**	2.466	1.187–5.124	**0.016**
eGFR, mL/min/1.73 m^2^	0.989	0.962–1.017	0.442	0.991	0.957–1.027	0.630
**Concomitant medication**						
Beta Blocker	0.716	0.187–2.733	0.625	0.607	0.110–3.340	0.566
ACE inhibitor	0.868	0.173–4.347	0.863	1.024	0.180–5.816	0.979
Angiotensin receptor blocker	1.044	0.260–4.184	0.952	0.1756	0.391–7.893	0.463
**Number of diuretic agents**	1.276	0.619–2.630	0.509	5.932	1.217–28.906	**0.028**
**Invasive hemodynamic parameters**						
**Mean pulmonary arterial pressure, mmHg**	1.177	1.049–1.321	**0.005**	1.130	1.006–1.269	**0.040**
Right atrial pressure, mmHg	1.124	0.992–1.273	0.067	1.057	0.926–1.205	0.411
Pulmonary artery wedge pressure, mmHg	1.116	0.995–1.253	0.061	1.045	0.924–1.182	0.484
Cardiac index, L/min/m^2^	0.920	0.338–2.502	0.870	0.656	0.248–1.739	0.397
Stroke volume index, mL/m^2^	0.974	0.920–1.031	0.369	0.950	0.890–1.014	0.124
**Pulmonary vascular resistance, dyn·s·cm^−5^**	1.005	0.999–1.010	0.086	1.010	1.000–1.020	**0.046**
Diastolic pressure gradient, mmHg	0.948	0.795–1.130	0.552	1.036	0.851–1.260	0.727
**Cardiac magnetic resonance imaging parameters**						
MOLLI-ECV, %	1.064	1.001–1.131	**0.045**	1.044	0.980–1.111	0.181
Left atrial area, cm^2^	1.120	0.993–1.264	0.065	1.081	0.940–1.241	0.274
Right atrial area, cm^2^	1.012	0.927–1.104	0.792	1.010	0.901–1.132	0.870
Left ventricular ejection fraction, %	0.981	0.921–1.045	0.554	1.031	0.939–1.133	0.524
Left ventricular end-diastolic volume index, mL/m^2^ (IQR)	1.016	0.974–1.060	0.468	0.984	0.932–1.040	0.569
Interventricular septum, mm	1.058	0.884–1.267	0.536	1.005	0.851–1.188	0.950
Right ventricular ejection fraction, %	0.973	0.924–1.025	0.297	0.990	0.931–1.053	0.757
Right ventricular end-diastolic volume index, mL/m^2^ (IQR)	1.009	0.993–1.026	0.260	1.022	0.975–1.071	0.370
**Transthorathic echocardiography parameters**						
Significant aortic valve stenosis	1.664	0.191–14.515	0.645	5.781	0.417–80.052	0.191
Significant aortic valve regurgitation	1.664	0.191–14.515	0.645	5.781	0.417–80.052	0.191
Significant mitral valve regurgitation	1.118	0.314–3.982	0.864	0.230	0.044–1.197	0.081

New York Heart Association (NYHA); N-terminal prohormone of brain natriuretic peptide (NT-proBNP); estimated glomerular filtration rate (eGFR); angiotensin converting enzyme (ACE); modified Look–Locker inversion recovery sequence derived extracellular volume (MOLLI-ECV). Valvular stenosis or regurgitation ≥ moderate was considered significant. *NT-proBNP was graded into quartiles. †Troponin T was graded into quartiles. Bold indicates statistical significance.

**Table 3:** Cox regression analysis for the cardiac light-chain amyloidosis cohort.

Variable	Crude Hazard Ratio	95% Confidence Interval	*p* Value	Adjusted Hazard Ratio	95% Confidence Interval	*p* Value
	Univariable Regression			Multivariable Regression		
**Clinical parameters**						
Age, years	0.983	0.942–1.026	0.432	0.972	0.929–1.018	0.233
Sex, male gender	0.632	0.249–1.603	0.334	0.491	0.173–1.398	0.183
NYHA functional class ≥ III	14.201	1.833–110.010	**0.011**	7.763	0.729–82.669	0.090
Systolic blood pressure, mmHg	0.981	0.962–0.999	**0.042**	0.992	0.971–1.013	0.441
Diastolic blood pressure, mmHg	0.988	0.949–1.029	0.568	0.983	0.949–1.018	0.328
NT-proBNP, pg/mL *	3.131	1.619–6.778	**0.001**	2.856	1.277–6.388	**0.011**
Troponin t, ng/mL †	1.894	1.171–2.778	**0.007**	1.180	0.740–1.880	0.487
eGFR, mL/min/1.73 m^2^	0.994	0.974–1.015	0.600	0.978	0.941–1.016	0.258
**Concomitant medication**						
Beta Blocker	1.106	0.444–2.756	0.828	1.132	0.408–3.139	0.811
ACE inhibitor	1.540	0.554–4.279	0.408	3.039	0.826–11.178	0.094
Angiotensin receptor blocker	0.660	0.220–1.977	0.457	1.125	0.351–3.608	0.843
Number of diuretic agents	1.185	0.759–1.850	0.455	1.456	0.777–2.728	0.241
**Invasive hemodynamic parameters**						
Mean pulmonary arterial pressure, mmHg	1.027	0.991–1.063	0.146	0.999	0.962–1.038	0.970
Right atrial pressure, mmHg	1.027	0.971–1.087	0.348	1.020	0.956–1.089	0.553
Pulmonary artery wedge pressure, mmHg	1.060	0.993–1.131	0.081	1.039	0.966–1.118	0.302
Cardiac index, L/min/m^2^	0.608	0.329–1.125	0.113	0.859	0.408–1.809	0.690
Stroke volume index, mL/m^2^	0.958	0.920–0.997	**0.034**	0.987	0.944–1.032	0.579
Pulmonary vascular resistance, dyn·s·cm^−5^	1.001	0.999–1.003	0.263	1.000	0.997–1.002	0.645
Diastolic pressure gradient, mmHg	1.042	0.965–1.126	0.292	0.978	0.891–1.073	0.635
**Cardiac magnetic resonance imaging parameters**						
MOLLI-ECV, %	1.042	1.011–1.073	**0.007**	1.012	0.970–1.055	0.590
Left atrial area, cm^2^	0.956	0.902–1.012	0.123	1.000	0.894–1.118	0.993
Right atrial area, cm^2^	0.943	0.876–1.016	0.122	1.004	0.919–1.097	0.922
Left ventricular ejection fraction, %	1.013	0.975–1.054	0.504	1.018	0.974–1.065	0.425
Left ventricular end-diastolic volume index, mL/m^2^ (IQR)	0.977	0.955–0.999	**0.041**	0.975	0.946–1.004	0.090
Interventricular septum, mm	1.209	1.057–1.384	**0.006**	1.099	0.948–1.273	0.211
Right ventricular ejection fraction, %	1.003	0.964–1.044	0.879	1.027	0.978–1.078	0.287
Right ventricular end-diastolic volume index, mL/m^2^ (IQR)	0.980	0.955–1.006	0.133	0.989	0.962–1.016	0.428
**Transthorathic echocardiography parameters**						
Significant mitral valve regurgitation	1.477	0.572–3.812	0.420	1.117	0.374–3.332	0.843

New York Heart Association (NYHA); N-terminal prohormone of brain natriuretic peptide (NT-proBNP); estimated glomerular filtration rate (eGFR); angiotensin converting enzyme (ACE); modified Look–Locker inversion recovery sequence derived extracellular volume (MOLLI-ECV). Valvular stenosis or regurgitation ≥ moderate was considered significant. * NT-proBNP was graded into quartiles. † Troponin T was graded into quartiles. Bold indicates statistical significance.

## Supplementary Materials

The following are available online at https://www.mdpi.com/2077-0383/9/4/1093/s1, Figure S1: Kaplan–Meier curves for event-free survival stratified by median mean pulmonary arterial pressure (mPAP) for the total cardiac amyloidosis cohort, Table S1: Cox regression analysis for the total study cohort.
