# Supplementary material for: Hemodynamic Profiles and Their Prognostic Relevance in Cardiac Amyloidosis
Source: J Clin Med. 2020 Apr 11;9(4):1093. doi: 10.3390/jcm9041093 (PMC7230541; doi:10.3390/jcm9041093)
Supplement: Supplementary file 1 [file jcm-09-01093-s001.pdf]

Table S1. Cox regression analysis for the total study cohort.

Variable	Crude hazard ratio	95% Confidence interval	*P* value	Adjusted hazard ratio	95% Confidence interval	*P* value
	**Univariable regression**			**Multivariable regression**		
**Clinical parameters**						
**Age, years**	0.966	0.931–1.003	0.072	0.958	0.924–0.993	**0.020**
**Sex, male gender**	0.525	0.255–1.083	0.081	0.277	0.122–0.628	**0.002**
**NYHA functional class ≥ III**	4.807	1.901–12.155	**0.001**	3.214	1.211–8.529	**0.019**
Systolic blood pressure, mmHg	0.979	0.961–0.998	**0.026**	0.989	0.971–1.008	0.250
Diastolic blood pressure, mmHg	0.985	0.952–1.019	0.368	0.988	0.959–1.018	0.421
**NT-proBNP, pg/mL***	2.103	1.452–3.047	**<0.001**	1.940	1.327–2.838	**0.001**
**Troponin t, ng/mL†**	1.694	1.213–2.365	**0.002**	1.558	1.073–2.264	**0.020**
eGFR, mL/min/1.73m^2	0.995	0.980–1.012	0.584	1.005	0.989–1.022	0.527
**Concomitant medication**						
Beta Blocker	0.928	0.446–1.931	0.841	0.777	0.356–1.697	0.527
ACE inhibitor	1.125	0.477–2.653	0.788	1.123	0.469–2.690	0.794
Angiotensin receptor blocker	0.711	0.304–1.665	0.432	0.693	0.294–1.634	0.402
Number of diuretic agents	1.351	0.924–1.976	0.121	1.501	0.923–2.440	0.101
**Invasive hemodynamic parameters**						
**Mean pulmonary arterial pressure, mmHg**	1.058	1.025–1.092	**<0.001**	1.034	1.001–1.069	**0.045**
Right atrial pressure, mmHg	1.065	1.010–1.122	**0.020**	1.048	0.987–1.112	0.125
**Pulmonary artery wedge pressure, mmHg**	1.101	1.038–1.166	**0.001**	1.073	1.011–1.139	**0.021**
Cardiac index, L/min/m^2	0.781	0.460–1.326	0.359	1.017	0.618–1.676	0.946
Stroke volume index, mL/m^2	0.972	0.943–1.002	0.070	0.984	0.955–1.014	0.288
Pulmonary vascular resistance, dyn·s·cm^{-5}	1.002	1.000–1.003	0.050	1.001	0.999–1.002	0.533
Diastolic pressure gradient, mmHg	1.004	0.917–1.099	0.930	0.984	0.905–1.070	0.711
**Cardiac magnetic resonance imaging parameters**						
MOLLI-ECV, %	1.051	1.022–1.080	**0.001**	1.018	0.987–1.049	0.261
Left atrial area, cm^2	0.992	0.946–1.040	0.734	0.993	0.936–1.053	0.806
Right atrial area, cm^2	0.952	0.904–1.002	0.058	0.950	0.899–1.003	0.065
Left ventricular ejection fraction, %	1.015	0.982–1.049	0.389	1.032	0.998–1.069	0.068
**Left ventricular end-diastolic volume index, mL/m^2 (IQR)**	0.977	0.959–0.995	**0.012**	0.972	0.952–0.992	**0.007**

Interventricular septum, mm	1.039	0.951–1.136	0.398	0.976	0.895–1.066	0.594
Right ventricular ejection fraction, %	0.995	0.966–1.025	0.764	1.020	0.986–1.056	0.245
Right ventricular end-diastolic volume index, mL/m² (IQR)	0.994	0.976–1.012	0.518	0.995	0.974–1.016	0.624
**Transthorathic echocardiography parameters**						
Significant aortic valve stenosis	1.072	0.142–8.109	0.946	6.110	0.628–59.402	0.119
Significant aortic valve regurgitation	1.072	0.142–8.109	0.946	6.110	0.628–59.402	0.119
Significant mitral valve regurgitation	1.116	0.529–2.353	0.773	0.549	0.249–1.212	0.138

NYHA indicates New York Heart Association; NT-proBNP, N-terminal prohormone of brain natriuretic peptide; eGFR, estimated glomerular filtration rate; ACE, angiotensin converting enzyme; MOLLI-ECV, modified Look-Locker inversion recovery sequence derived extracellular volume. Valvular stenosis or regurgitation ≥ moderate was considered significant. *NT-proBNP was graded into quartiles. †Troponin T was graded into quartiles. **Bold** indicates statistical significance.

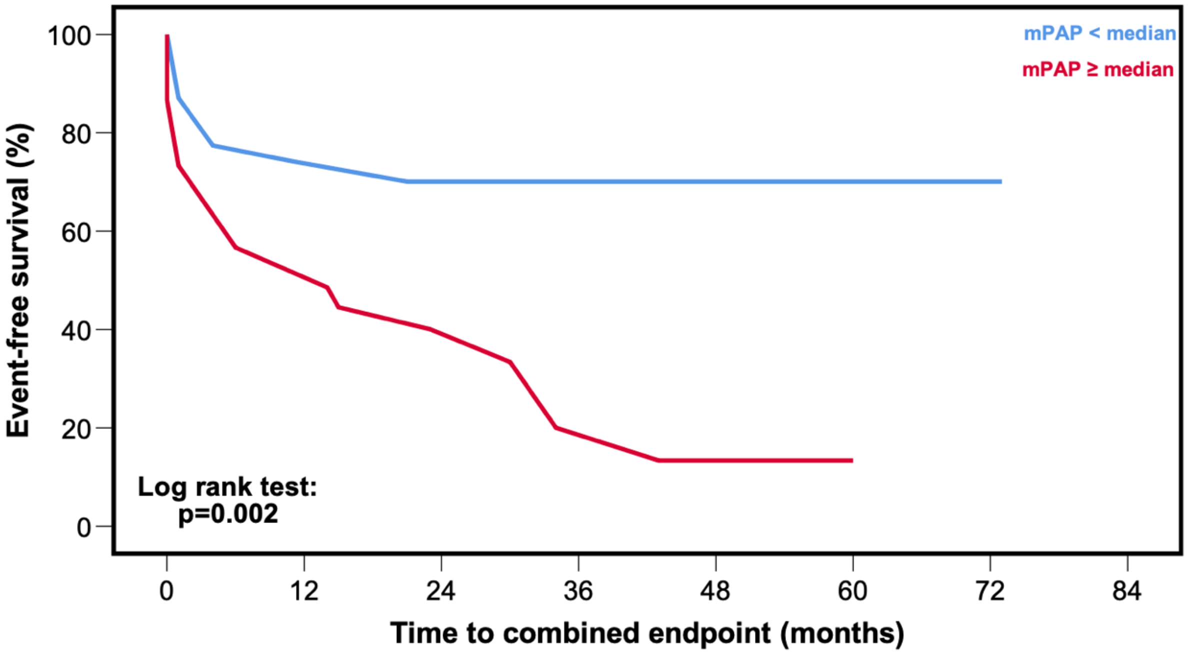

Figure S1. Kaplan-Meier curves for event-free survival stratified by median mean pulmonary arterial pressure (mPAP) for the total cardiac amyloidosis cohort.
